# Data on bioactive peptides derived from chicken hydrolysate with potential alcohol dehydrogenase stabilizing activity and *in silico* analysis of their potential activity and applicability

**DOI:** 10.1016/j.dib.2020.105163

**Published:** 2020-02-03

**Authors:** Chuqiao Xiao, Mouming Zhao, Feibai Zhou, Marta Gallego, Fidel Toldrá, Leticia Mora

**Affiliations:** a School of Food Science and Engineering, South China University of Technology, Guangzhou 510640, China; b Guangdong Food Green Processing and Nutrition Regulation Technology Research Center, Guangzhou 510640, China; c Instituto de Agroquímica y Tecnología de Alimentos (CSIC), Avenue Agustín Escardino 7, 46980 Paterna, Valencia, Spain

**Keywords:** Bioactive peptides, Chicken breast, Alcohol dehydrogenase stabilizing, LC-MS/MS, *In silico* analysis

## Abstract

Bioactive peptides have attracted extensive attention worldwide as natural alternatives to promote human health and wellness. Previous studies have shown that chicken hydrolysates could enhance alcohol dehydrogenase, and subsequently they facilitate alcohol metabolism and ameliorate alcohol-induced liver injury. The data presented in this article support the accompanying research article “Isolation and identification of alcohol dehydrogenase stabilizing peptides from Alcalase digested chicken breast hydrolysates”. Present article details all 82 peptides identified from the most active fractions of chicken hydrolysates, and 154 peptides from *in silico* digestion of the 82 identified peptides, together with the prediction of their potential bioactivity and applicability using several *in silico* assays.

**Table:** Specifications Table

Subject	Food science, Food biochemistry
Specific subject area	Bioactive peptides
Type of data	Table
How data were acquired	The Alcalase digested chicken hydrolysate was separated through size-exclusion chromatography (SEC) and reversed-phase high-performance liquid chromatography (RP-HPLC) before the identification using nano-LC and ESI-Q-ToF in tandem mass spectrometry. Peptides were sequenced through database searching using Mascot. The identified peptides were further analyzed using various *in silico* methods including ExPASy PeptideCutter tool, Peptide Ranker, AllerTOP, and ToxinPred.
Data format	Analyzed
Parameters for data collection	MS: Positive polarity mode. MS1 scan from 350 to 1250 *m*/*z* for 250 ms, MS2 scan from 100 to 1500 *m*/*z* for 50 ms. Ions charging 1 to 5. Mascot data analysis: Significance threshold *p* < 0.05, using Chordata taxonomy, none enzyme digestion, and Uniprot database. The tolerance on the mass measurement was 0.3 Da for MS1 and 100 ppm for MS2.
Description of data collection	Data analysis was done using ExPASy PeptideCutter tool (http://web.expasy.org/peptide_cutter/) to simulate the gastrointestinl digestion. The potential bioactivity was predicted using the Peptide Ranker software (http://distilldeep.ucd.ie/PeptideRanker/). The potential peptide allergenicity was predicted using the AllerTOP v. 2.0 software (http://www.ddg-pharmfac.net/AllerTOP/index.html). Peptide toxicity and physicochemical properties (i.e., hydrophobicity, amphipathicity, steric hindrance, and pI) were studied using the ToxinPred software (http://crdd.osdd.net/raghava/toxinpred/).
Data source location	Instituto de Agroquímica y Tecnología de Alimentos (CSIC), Valencia, Spain
Data accessibility	Within this article.
Related research article	Author's name: Chuqiao Xiao^1,2,3^, Mouming Zhao^1,2^, Feibai Zhou^1,2^, Marta Gallego^3^, Jie Gao^1,2^, Fidel Toldrá^3^, and Leticia Mora^3^* Title: Isolation and identification of alcohol dehydrogenase stabilizing peptides from Alcalase digested chicken breast hydrolysates Journal: Journal of Functional Foods DOI: https://doi.org/10.1016/j.jff.2019.103617

**Table:** 

**Value of the Data** • The data in this article includes a series of bioactive peptides separated and identified from a chicken hydrolysate that has been reported to exert alcohol dehydrogenase stabilizing activity. The potential bioactivity and applicability have been assessed and listed in this article. • This article can benefit those who are interested in the separation and identification of bioactive peptides from food protein, with potential protection against alcoholic liver injury. • The data can provide some useful information about the amino acid composition of the potential bioactive peptides, which is important for the quantitative-structure activity relationship (QSAR) study of bioactive peptides. Also, some of the peptides are assessed to be neither bioactive nor physically stable, and this information could result useful in future research.

## 1 Data

The data in this article include a total 82 peptides from the most active fractions of a chicken hydrolysate after SEC and RP-HPLC separation. Raw data is showed in Table 1 of Supplementary material, including search parameters. Table 1 lists all these peptides together with the potential bioactivity and applicability, indicated by Peptide Ranker score, potential peptide allergenicity, toxicity and physicochemical properties (i.e., hydrophobicity, amphipathicity, steric hindrance, pI, and molecular weight). Also, as potential functional food ingredients, peptides must be digested and absorbed through the gastrointestinal tract to exert activity. Hence, all peptides were further subjected to *in silico* gastrointestinal digestion and a total of 154 peptides were generated. All the 154 peptides are listed in Table 2, as well as their potential bioactivity and applicability.

**Table 1:** Identified peptides and *in silico* prediction of their allergenicity, toxicity, and physicochemical properties.

No.	Peptide Sequence	Peptide Ranker Score	Allergenicity Prediction	Nearest Protein	Toxicity Prediction	SVM Score	Steric Hindrance	Amphipa thicity	Hydropho bicity	pI	Molecular Weight (Da)
1	DPDDFPL	0.91	non-allergen	UniProtKB accession number P17676	Non-Toxin	−0.88	0.6	0	−0.17	3.43	817.35
2	NKISVVGVGAVGMACAISILMKDLA	0.86	non-allergen	UniProtKB accession number Q10ST8	Non-Toxin	−1.47	0.65	0.29	0.13	8.54	2459.33
3	DPQYPPGPPAFP	0.85	non-allergen	UniProtKB accession number Q9S8M0	Non-Toxin	−0.27	0.52	0.1	−0.07	3.8	1281.60
4	ADGPLKGIL	0.77	non-allergen	UniProtKB accession number Q9NP55	Non-Toxin	−0.72	0.6	0.41	0.05	6.19	882.52
5	RDPQYPPGPPAFP	0.76	non-allergen	UniProtKB accession number Q9S8M0	Non-Toxin	−0.35	0.53	0.28	−0.2	6.19	1437.70
6	ARDPQYPPGPPAFP	0.72	non-allergen	UniProtKB accession number Q9S8M0	Non-Toxin	−0.38	0.53	0.26	−0.16	6.19	1508.74
7	LPVPAFNVINGGSHAGNKL	0.70	non-allergen	UniProtKB accession number Q8H0K8	Non-Toxin	−1.14	0.59	0.27	0.03	9.11	1904.03
8	ENPKKYIPGTKMIFAGIK	0.69	non-allergen	UniProtKB accession number Q9SSX0	Non-Toxin	−0.89	0.64	0.89	−0.13	9.84	2034.13
9	AGFGGDDAPRAVFP	0.69	non-allergen	UniProtKB accession number Q8WYQ3	Non-Toxin	−1.19	0.62	0.18	−0.03	4.21	1375.65
10	GFAGDDAPRAVFP	0.67	non-allergen	UniProtKB accession number Q00587	Non-Toxin	−1.16	0.61	0.19	−0.04	4.21	1318.63
11	DGPLKGIL	0.66	non-allergen	UniProtKB accession number P09564	Non-Toxin	−0.76	0.62	0.46	0.03	6.19	811.48
12	ATGNPNPDIVWLK	0.60	non-allergen	UniProtKB accession number Q96N21	Non-Toxin	−0.58	0.6	0.28	−0.06	6.19	1423.75
13	PPGKPGPPGPPGPPGIQGIHQTL	0.55	non-allergen	UniProtKB accession number Q9S8M0	Non-Toxin	−0.15	0.51	0.33	−0.03	9.11	2195.19
14	FDEKPADLPSL	0.53	non-allergen	UniProtKB accession number P23582	Non-Toxin	−1.19	0.58	0.45	−0.15	4.03	1230.61
15	AVNDPFID	0.49	non-allergen	UniProtKB accession number P86071	Non-Toxin	−1.03	0.66	0	0	3.57	889.42
16	DVPGPVLDLKPV	0.47	non-allergen	UniProtKB accession number Q5T440	Non-Toxin	−1.23	0.59	0.31	0.01	4.21	1247.71
17	VIDVPGPVRNL	0.45	non-allergen	UniProtKB accession number Q7M2H1	Non-Toxin	−0.6	0.63	0.22	−0.02	6.19	1177.68
18	VNVLDKPGPPAAF	0.42	non-allergen	UniProtKB accession number Q5T0Z8	Non-Toxin	−1.16	0.59	0.28	0.02	6.19	1323.72
19	FDEKPADLPSLVE	0.39	non-allergen	UniProtKB accession number P23582	Non-Toxin	−1.16	0.6	0.48	−0.13	3.92	1458.72
20	GGFAPNILDNHEALE	0.37	non-allergen	UniProtKB accession number Q9C0K0	Non-Toxin	−1.23	0.59	0.27	−0.03	4.14	1595.76
21	AEEEFPDL	0.33	non-allergen	UniProtKB accession number Q9BTT0	Non-Toxin	−0.63	0.61	0.48	−0.16	3.51	948.41
22	FMVLPVGAA	0.30	non-allergen	UniProtKB accession number P69448	Non-Toxin	−1.07	0.61	0	0.34	5.88	903.49
23	PFQSSASSPSPSKNE	0.30	non-allergen	UniProtKB accession number Q8WWM7	Non-Toxin	−0.89	0.55	0.41	−0.26	6.35	1548.71
24	DTFTTPGPPYAL	0.29	non-allergen	UniProtKB accession number P54259	Non-Toxin	−0.54	0.55	0	0.01	3.8	1278.61
25	IEDPFDQDDWE	0.28	non-allergen	UniProtKB accession number Q9SLY8	Non-Toxin	−0.69	0.67	0.34	−0.29	3.26	1407.55
26	MVVDGVKLM	0.25	non-allergen	UniProtKB accession number P48494	Non-Toxin	−1.03	0.7	0.41	0.11	6.19	990.52
27	NFPTYDGKDRVIDL	0.23	non-allergen	UniProtKB accession number Q9H0F7	Non-Toxin	−1.15	0.66	0.44	−0.24	4.43	1651.82
28	DNHEALELL	0.23	non-allergen	UniProtKB accession number Q9S8F6	Non-Toxin	−1.14	0.55	0.44	−0.13	4.14	1052.51
29	EAEEFLPD	0.21	non-allergen	UniProtKB accession number A2WMG6	Non-Toxin	−0.72	0.61	0.48	−0.16	3.51	948.41
30	EDLQKPVLDL	0.19	non-allergen	UniProtKB accession number P02647	Non-Toxin	−1.02	0.62	0.62	−0.18	4.03	1168.63
31	FDPVVEEKI	0.15	non-allergen	UniProtKB accession number Q9S8N3	Non-Toxin	−1.17	0.66	0.69	−0.08	4.14	1074.56
32	VPEDSQEECAITY	0.15	non-allergen	UniProtKB accession number Q84T68	Non-Toxin	−0.79	0.63	0.39	−0.17	3.51	1482.62
33	DISNADRLGFSEVEQVQ	0.10	non-allergen	UniProtKB accession number Q99766	Non-Toxin	−1.42	0.66	0.44	−0.21	3.92	1905.91
34	RDKETPSGFTLDDVIQT	0.10	non-allergen	UniProtKB accession number Q5SW96	Non-Toxin	−1.42	0.63	0.51	−0.27	4.23	1920.94
35	LTDTPTLASPEGSGET	0.07	non-allergen	UniProtKB accession number Q8WYQ3	Non-Toxin	−1.53	0.56	0.16	−0.11	3.58	1574.73
36	SEEEDNEEEAEV	0.05	non-allergen	UniProtKB accession number Q9BTT0	Non-Toxin	−0.85	0.67	0.74	−0.43	3.32	1407.52
37	NVLIFDLGGGTFDVSILTIDDGIFEVK	0.02	non-allergen	UniProtKB accession number Q53IQ4	Non-Toxin	−1.63	0.67	0.18	0.1	3.72	2896.51
38	PATIVPIDEESRNGTILVDNMLIKGTAAGPDPTIE	0.02	non-allergen	UniProtKB accession number Q6UX39	Non-Toxin	−0.86	0.62	0.28	−0.06	4.02	3646.88
39	RIPVVLPEDEGIYT	0.15	allergen	UniProtKB accession number Q6XNP7	Non-Toxin	−1.7	0.63	0.36	−0.06	4.14	1599.85
40	ELGPMTKPLCLKAA	0.61	allergen	NCBI gi nimber 6069656	Non-Toxin	−1.41	0.57	0.61	−0.04	8.54	1470.79
41	LEQNQPIDDMIPA	0.32	allergen	UniProtKB accession number Q01412	Non-Toxin	−1.39	0.64	0.29	−0.13	3.5	1482.70
42	AYEPVWAIGTGKTATPQ	0.22	allergen	UniProtKB accession number P85814	Non-Toxin	−1.28	0.58	0.36	−0.02	6.35	1788.90
43	GEHGDSSVPVWSGVNVA	0.35	allergen	UniProtKB accession number O42799	Non-Toxin	−1.26	0.59	0.16	0	4.36	1695.79
44	DLFDPVIQD	0.18	allergen	NCBI gi nimber 423193	Non-Toxin	−1.25	0.66	0.14	−0.06	3.43	1060.51
45	VPTQHRGPVVL	0.18	allergen	NCBI gi nimber 741844	Non-Toxin	−1.23	0.54	0.47	−0.08	10.11	1201.69
46	AVGAVFDISNADRL	0.39	allergen	NCBI gi nimber 1171011	Non-Toxin	−1.2	0.65	0.18	−0.02	4.21	1446.75
47	AVGAVFDIS	0.22	allergen	NCBI gi nimber 29163773	Non-Toxin	−1.2	0.65	0	0.23	3.8	877.45
48	FPFDVPSEPK	0.58	allergen	NCBI gi nimber 63052	Non-Toxin	−1.18	0.58	0.49	−0.12	4.38	1161.57
49	LPTGIPIVYE	0.19	allergen	NCBI gi nimber 50199132	Non-Toxin	−1.08	0.59	0.13	0.18	4	1100.61
50	KDLFDPVIQ	0.27	allergen	NCBI gi nimber 1836010	Non-Toxin	−1.07	0.65	0.55	−0.1	4.21	1073.58
51	GDLGIEIPAE	0.18	allergen	NCBI gi nimber 542132	Non-Toxin	−1.06	0.63	0.25	0.05	3.58	1012.51
52	SNKKPELIDML	0.46	allergen	NCBI gi nimber 3004471	Non-Toxin	−1.04	0.64	0.78	−0.22	6.42	1286.69
53	VPAFNVINGGSHAGNKL	0.60	allergen	UniProtKB accession number B0Y665	Non-Toxin	−1.03	0.6	0.3	0.01	9.11	1693.89
54	DLFDPVIQ	0.31	allergen	NCBI gi nimber 1836010	Non-Toxin	−1.02	0.65	0.16	0.03	3.57	945.48
55	DLAGRDLTDYLM	0.36	allergen	UniProtKB accession number Q28049	Non-Toxin	−0.99	0.65	0.2	−0.15	3.94	1381.65
56	TLVDVVEDKLKGE	0.06	allergen	NCBI gi nimber 14148979	Non-Toxin	−0.98	0.66	0.76	−0.17	4.32	1443.78
57	LLLLAPGH	0.38	allergen	UniProtKB accession number P01315	Non-Toxin	−0.96	0.46	0.18	0.26	7.1	832.52
58	YGKDATNVGDEGGFAPNIL	0.32	allergen	NCBI gi nimber 160347126	Non-Toxin	−0.94	0.65	0.26	−0.06	4.03	1936.92
59	NDEELNKLLGKVT	0.20	allergen	NCBI gi nimber 47117355	Non-Toxin	−0.94	0.65	0.76	−0.26	4.68	1471.79
60	HLDIPKML	0.66	allergen	UniProtKB accession number P35776	Non-Toxin	−0.92	0.54	0.64	−0.03	7.09	965.54
61	IDDHFLFDKPVSP	0.46	allergen	NCBI gi nimber 3914446	Non-Toxin	−0.92	0.58	0.39	−0.08	4.42	1528.76
62	NGDKKSLGELIHTL	0.39	allergen	NCBI gi nimber 261865475	Non-Toxin	−0.92	0.59	0.72	−0.17	7.1	1523.83
63	INDPFIDLN	0.60	allergen	NCBI gi nimber 7228147	Non-Toxin	−0.9	0.67	0	−0.02	3.57	1059.52
64	IDDHFLFDKPVSPL	0.71	allergen	NCBI gi nimber 3914446	Non-Toxin	−0.89	0.58	0.37	−0.04	4.42	1641.84
65	DDHFLFDKPVSPL	0.75	allergen	NCBI gi nimber 3914446	Non-Toxin	−0.87	0.57	0.39	−0.1	4.42	1528.76
66	NYILDHLLGSK	0.29	allergen	UniProtKB accession number P00709	Non-Toxin	−0.87	0.58	0.47	−0.06	7.09	1271.69
67	VSHRSGETEDTFIADL	0.10	allergen	NCBI gi nimber 60116876	Non-Toxin	−0.87	0.59	0.4	−0.18	4.31	1775.83
68	DSSTNGLISFIK	0.32	allergen	UniProtKB accession number Q39869	Non-Toxin	−0.83	0.64	0.31	−0.06	6.19	1280.66
69	VHVLLVNPHTGAT	0.18	allergen	NCBI gi nimber 5689675	Non-Toxin	−0.82	0.5	0.22	0.09	7.26	1356.75
70	GPPDPILGVT	0.83	allergen	UniProtKB accession number Q9UMD9	Non-Toxin	−0.81	0.57	0	0.1	3.8	964.52
71	DQIDDEIKLI	0.25	allergen	NCBI gi nimber 28374072	Non-Toxin	−0.8	0.69	0.62	−0.18	3.84	1200.62
72	DNPVDLI	0.38	allergen	NCBI gi nimber 105969543	Non-Toxin	−0.79	0.65	0	−0.05	3.57	784.40
73	AKYGKDATNVGDEGGFAPNIL	0.28	allergen	NCBI gi nimber 160347126	Non-Toxin	−0.76	0.65	0.41	−0.09	4.56	2136.05
74	EVMIDVLK	0.14	allergen	NCBI gi nimber 19039	Non-Toxin	−0.71	0.69	0.62	0.02	4.38	945.52
75	DQIDDEIKLIGY	0.30	allergen	UniProtKB accession number P02635	Non-Toxin	−0.66	0.69	0.52	−0.14	3.84	1420.71
76	DDVIQTGVDNPGHPFIM	0.43	allergen	NCBI gi nimber 7228147	Non-Toxin	−0.53	0.62	0.16	−0.03	3.93	1853.86
77	SLKPEFVDIINAKH	0.40	allergen	UniProtKB accession number Q86D83	Non-Toxin	−0.42	0.59	0.72	−0.11	7.1	1609.88
78	LKPEFVDIINAKH	0.28	allergen	UniProtKB accession number Q86D83	Non-Toxin	−0.42	0.6	0.77	−0.1	7.1	1522.85
79	NVMSGGTTMYPGIADRM	0.18	allergen	NCBI gi nimber 62550933	Non-Toxin	−0.32	0.66	0.14	−0.06	6.19	1799.80
80	GVDNPGHPYIM	0.69	allergen	NCBI gi nimber 14423730	Non-Toxin	−0.22	0.59	0.13	0	5.09	1198.54
81	PDPLDPTCSLCTCEEGSMRCQKKPC	0.80	non-allergen	UniProtKB accession number P08603	Toxin	0.03	0.59	0.54	−0.27	4.79	2740.15
82	IPGPPTGPIKF	0.88	non-allergen	UniProtKB accession number Q9S8M0	Toxin	0.09	0.56	0.33	0.08	9.11	1122.64

**Table 2:** Peptides obtained from the *in silico* gastrointestinal digestion simulation and prediction of their bioactivity, allergenicity, toxicity, and physicochemical properties.

No.	Peptide Sequence	Peptide Ranker Score	Allergenicity Prediction	Nearest Protein	Toxicity Prediction	SVM Score	Steric Hindrance	Amphipa thicity	Hydropho bicity	pI	Molecular Weight (Da)
1	IF	0.95	non-allergen	UniProtKB accession number Q8WW43	Non-Toxin	−0.8	0.7	0	0.67	5.88	278.37
2	GPPDPIL	0.91	non-allergen	UniProtKB accession number Q9Y2D1	Non-Toxin	−0.24	0.54	0	0.07	3.8	707.92
3	GG	0.89	non-allergen	UniProtKB accession number Q04130	Non-Toxin	−0.8	0.68	0	0.16	5.88	132.14
4	DPQYPPGPPAF	0.88	non-allergen	UniProtKB accession number Q9S8M0	Non-Toxin	−0.27	0.53	0.11	−0.07	3.8	1185.44
5	QKPVL	0.82	non-allergen	UniProtKB accession number P49918	Non-Toxin	−0.88	0.59	0.98	−0.16	9.11	583.8
6	KPC	0.71	non-allergen	UniProtKB accession number P01075	Non-Toxin	−0.72	0.55	1.22	−0.38	8.57	346.47
7	PEDEGI	0.70	non-allergen	UniProtKB accession number Q9S8M0	Non-Toxin	−0.9	0.64	0.42	−0.19	3.58	658.74
8	APGH	0.65	non-allergen	UniProtKB accession number Q9S8K1	Non-Toxin	−0.86	0.39	0.36	−0.02	7.1	380.45
9	GIL	0.61	non-allergen	UniProtKB accession number O14569	Non-Toxin	−0.79	0.64	0	0.47	5.88	301.43
10	AVF	0.59	non-allergen	UniProtKB accession number P21926	Non-Toxin	−0.83	0.64	0	0.47	5.88	335.43
11	PDP	0.57	non-allergen	UniProtKB accession number Q9T2Q0	Non-Toxin	−0.82	0.49	0	−0.29	3.8	327.36
12	GGDDAPR	0.56	non-allergen	UniProtKB accession number Q7M1V9	Non-Toxin	−1.07	0.63	0.35	−0.39	4.21	686.76
13	KPEF	0.56	non-allergen	UniProtKB accession number A6N0M9	Non-Toxin	−0.89	0.6	1.23	−0.3	6.35	519.64
14	ADGP	0.56	non-allergen	UniProtKB accession number P83184	Non-Toxin	−0.71	0.58	0	−0.09	3.8	358.39
15	GDSSVPVW	0.56	non-allergen	UniProtKB accession number P02747	Non-Toxin	−0.99	0.59	0	0.04	3.8	846.01
16	AGR	0.55	non-allergen	UniProtKB accession number P80806	Non-Toxin	−0.78	0.63	0.82	−0.45	10.11	302.36
17	AG	0.55	non-allergen	UniProtKB accession number Q109R6	Non-Toxin	−0.8	0.6	0	0.21	5.88	146.16
18	PTGIPIV	0.54	non-allergen	UniProtKB accession number Q9T2Q0	Non-Toxin	−0.81	0.58	0	0.26	5.88	695.96
19	AGDDAPR	0.54	non-allergen	UniProtKB accession number Q7M1V9	Non-Toxin	−1.09	0.61	0.35	−0.37	4.21	700.78
20	GGGT	0.53	non-allergen	UniProtKB accession number Q04130	Non-Toxin	−0.77	0.64	0	0.07	5.88	290.33
21	DVPGPVL	0.50	non-allergen	UniProtKB accession number P83184	Non-Toxin	−0.67	0.58	0	0.13	3.8	695.91
22	VDIINAK	0.40	non-allergen	UniProtKB accession number Q7M1V6	Non-Toxin	−0.38	0.69	0.52	−0.03	6.19	772.01
23	SGVNVA	0.39	non-allergen	UniProtKB accession number Q9S8V2	Non-Toxin	−0.98	0.65	0	0.1	5.88	545.67
24	VIDVPGPVR	0.34	non-allergen	UniProtKB accession number P46269	Non-Toxin	−0.65	0.63	0.27	−0.01	6.19	951.26
25	SNK	0.33	non-allergen	UniProtKB accession number Q7FAY6	Non-Toxin	−0.76	0.66	1.22	−0.67	9.11	347.4
26	DPTCS	0.31	non-allergen	UniProtKB accession number P01076	Non-Toxin	−0.88	0.56	0	−0.24	3.8	521.59
27	DR	0.29	non-allergen	UniProtKB accession number F2YHL2	Non-Toxin	−0.8	0.72	1.23	−1.24	6.19	289.3
28	ACAISI	0.29	non-allergen	UniProtKB accession number Q7M1U1	Non-Toxin	−0.98	0.6	0	0.29	5.85	576.78
29	ISVVGVGAVGM	0.29	non-allergen	UniProtKB accession number Q10ST8	Non-Toxin	−1.13	0.67	0	0.33	5.88	988.38
30	INDP	0.28	non-allergen	UniProtKB accession number P80819	Non-Toxin	−0.77	0.64	0	−0.17	3.8	457.53
31	IPGTK	0.27	non-allergen	UniProtKB accession number P00068	Non-Toxin	−0.95	0.59	0.73	−0.09	9.11	514.69
32	GTAAGPDPTIE	0.27	non-allergen	UniProtKB accession number Q96IK1	Non-Toxin	−0.84	0.57	0.12	−0.03	3.67	1028.23
33	AGIK	0.25	non-allergen	UniProtKB accession number Q109R6	Non-Toxin	−0.85	0.65	0.92	0.01	9.11	387.53
34	PVPAF	0.23	non-allergen	UniProtKB accession number P83184	Non-Toxin	−0.99	0.53	0	0.25	5.88	529.69
35	AIGTGK	0.22	non-allergen	UniProtKB accession number B8YI64	Non-Toxin	−0.78	0.63	0.61	0	9.11	545.72
36	VDNM	0.22	non-allergen	UniProtKB accession number Q8N0T1	Non-Toxin	−0.76	0.75	0	−0.14	3.8	477.58
37	CTCEEGSM	0.20	non-allergen	UniProtKB accession number P30569	Non-Toxin	−0.22	0.64	0.32	−0.15	3.8	859.05
38	AGNK	0.19	non-allergen	UniProtKB accession number Q7M1V9	Non-Toxin	−0.74	0.66	0.92	−0.33	9.11	388.47
39	DNPVD	0.19	non-allergen	UniProtKB accession number P80098	Non-Toxin	−0.72	0.67	0	−0.32	3.57	558.6
40	GSK	0.19	non-allergen	UniProtKB accession number Q13794	Non-Toxin	−0.74	0.63	1.22	−0.4	9.11	290.35
41	DISNADR	0.18	non-allergen	UniProtKB accession number P41208	Non-Toxin	−0.93	0.67	0.35	−0.45	4.21	789.88
42	DVPSEPK	0.17	non-allergen	UniProtKB accession number A0MH06	Non-Toxin	−1.03	0.58	0.71	−0.33	4.38	770.92
43	AVNDP	0.16	non-allergen	UniProtKB accession number P80819	Non-Toxin	−0.92	0.62	0	−0.13	3.8	514.59
44	IDDH	0.16	non-allergen	UniProtKB accession number Q9HBK9	Non-Toxin	−0.9	0.55	0.36	−0.28	4.2	498.54
45	DDH	0.16	non-allergen	UniProtKB accession number O00421	Non-Toxin	−0.82	0.51	0.48	−0.61	4.2	385.36
46	DSSTNG	0.16	non-allergen	UniProtKB accession number Q9S8W0	Non-Toxin	−0.59	0.63	0	−0.32	3.8	579.59
47	TGAT	0.15	non-allergen	UniProtKB accession number Q9T2R4	Non-Toxin	−0.78	0.56	0	0.01	5.88	348.4
48	DDVIQTGVDNPGHP	0.15	non-allergen	UniProtKB accession number Q96IK1	Non-Toxin	−0.53	0.6	0.19	−0.15	3.93	1463.73
49	GIEIPAE	0.14	non-allergen	UniProtKB accession number Q06I91	Non-Toxin	−0.88	0.62	0.36	0.08	3.8	727.91
50	IEDP	0.14	non-allergen	UniProtKB accession number P80819	Non-Toxin	−0.72	0.62	0.32	−0.17	3.67	472.54
51	DNH	0.14	non-allergen	UniProtKB accession number Q8WW43	Non-Toxin	−0.81	0.51	0.48	−0.59	5.09	384.38
52	IAD	0.14	non-allergen	UniProtKB accession number P86005	Non-Toxin	−0.81	0.66	0	0.09	3.8	317.37
53	IS	0.13	non-allergen	UniProtKB accession number P42055	Non-Toxin	−0.8	0.61	0	0.23	5.88	218.27
54	TTPGPPY	0.13	non-allergen	UniProtKB accession number A0MH06	Non-Toxin	−0.2	0.5	0	−0.06	5.88	731.89
55	DPVIQD	0.13	non-allergen	UniProtKB accession number Q9S8N3	Non-Toxin	−1.12	0.66	0.21	−0.15	3.57	685.81
56	VL	0.13	non-allergen	UniProtKB accession number P21730	Non-Toxin	−0.8	0.61	0	0.54	5.88	230.33
57	VDIINAK	0.12	non-allergen	UniProtKB accession number P80818	Non-Toxin	−0.38	0.69	0.52	−0.03	6.19	772.01
58	AVGAV	0.12	non-allergen	UniProtKB accession number A9UGV5	Non-Toxin	−0.8	0.62	0	0.35	5.88	415.55
59	ETPSG	0.11	non-allergen	UniProtKB accession number P26436	Non-Toxin	−0.99	0.56	0.25	−0.19	4	489.54
60	AEEEF	0.11	non-allergen	UniProtKB accession number Q9S8F6	Non-Toxin	−0.84	0.65	0.76	−0.2	3.68	623.67
61	DQDD	0.11	non-allergen	UniProtKB accession number P60006	Non-Toxin	−0.85	0.74	0.31	−0.71	3.43	491.45
62	DIS	0.11	non-allergen	UniProtKB accession number Q9C0F1	Non-Toxin	−0.82	0.66	0	−0.08	3.8	333.37
63	VPTQH	0.11	non-allergen	UniProtKB accession number Q9S8X3	Non-Toxin	−1.04	0.45	0.54	−0.16	7.1	580.71
64	ENPK	0.11	non-allergen	UniProtKB accession number P56615	Non-Toxin	−0.77	0.62	1.23	−0.61	6.35	486.57
65	AK	0.10	non-allergen	UniProtKB accession number Q9H246	Non-Toxin	−0.79	0.6	1.83	−0.43	9.11	217.28
66	EAL	0.09	non-allergen	UniProtKB accession number Q9S8F6	Non-Toxin	−0.84	0.58	0.42	0.05	4	331.4
67	GVT	0.09	non-allergen	UniProtKB accession number P31110	Non-Toxin	−0.84	0.64	0	0.17	5.88	275.34
68	EVM	0.09	non-allergen	UniProtKB accession number Q8NBU5	Non-Toxin	−0.79	0.72	0.42	0.06	4	377.49
69	TATPQ	0.09	non-allergen	UniProtKB accession number O00585	Non-Toxin	−0.91	0.52	0.25	−0.17	5.88	516.61
70	VSH	0.07	non-allergen	UniProtKB accession number Q9NWK9	Non-Toxin	−0.83	0.41	0.48	−0.04	7.1	341.4
71	IDV	0.07	non-allergen	UniProtKB accession number P86080	Non-Toxin	−0.8	0.72	0	0.18	3.8	345.43
72	VID	0.06	non-allergen	UniProtKB accession number Q08480	Non-Toxin	−0.8	0.72	0	0.18	3.8	345.43
73	VVDGVK	0.06	non-allergen	UniProtKB accession number P86008	Non-Toxin	−1.07	0.7	0.61	−0.01	6.19	615.81
74	DPVVEEK	0.06	non-allergen	UniProtKB accession number Q9S8N3	Non-Toxin	−1.02	0.65	0.89	−0.29	4.14	814.98
75	DT	0.05	non-allergen	UniProtKB accession number O75366	Non-Toxin	−0.8	0.65	0	−0.45	3.8	234.22
76	SEVEQVQ	0.05	non-allergen	UniProtKB accession number Q9BTT0	Non-Toxin	−1.11	0.66	0.72	−0.26	3.8	817.95
77	NVINGGSH	0.04	non-allergen	UniProtKB accession number Q99467	Non-Toxin	−0.68	0.6	0.18	−0.04	7.1	796.96
78	VDVVEDK	0.04	non-allergen	UniProtKB accession number A2XG55	Non-Toxin	−0.64	0.71	0.71	−0.22	4.03	802.97
79	NDEE	0.04	non-allergen	UniProtKB accession number P60006	Non-Toxin	−0.76	0.72	0.64	−0.65	3.58	505.48
80	NF	0.03	non-allergen	UniProtKB accession number Q8H9D6	Non-Toxin	−0.8	0.73	0	−0.02	5.88	279.31
81	SGETEDT	0.03	non-allergen	UniProtKB accession number Q9T2R4	Non-Toxin	−1.12	0.63	0.36	−0.35	3.58	737.76
82	EAEE	0.03	non-allergen	UniProtKB accession number Q9BTT0	Non-Toxin	−0.9	0.64	0.95	−0.4	3.68	476.48
83	EQNQPIDDM	0.27	allergen	UniProtKB accession number Q01412	Non-Toxin	−1.34	0.68	0.42	−0.35	3.5	1089.27
84	ASPEGSGET	0.14	allergen	NCBI gi nimber 285005079	Non-Toxin	−1.27	0.58	0.28	−0.16	3.8	833.92
85	PVGAA	0.31	allergen	NCBI gi nimber 1588669	Non-Toxin	−1.14	0.56	0	0.23	5.88	413.53
86	IPVVL	0.24	allergen	UniProtKB accession number P01504	Non-Toxin	−1.06	0.6	0	0.45	5.88	539.79
87	TDTPTL	0.04	allergen	UniProtKB accession number Q06478	Non-Toxin	−0.97	0.54	0	−0.13	3.8	646.77
88	EPVW	0.49	allergen	UniProtKB accession number P09944	Non-Toxin	−0.94	0.56	0.32	·	4	529.64
89	PGIADR	0.13	allergen	UniProtKB accession number Q7M278	Non-Toxin	−0.93	0.62	0.41	−0.24	6.19	627.77
90	KPEL	0.19	allergen	NCBI gi nimber 3004471	Non-Toxin	−0.92	0.56	1.23	−0.32	6.35	485.63
91	DPVIQ	0.17	allergen	NCBI gi nimber 7228147	Non-Toxin	−0.91	0.64	0.25	−0.04	3.8	570.71
92	DDVIQT	0.09	allergen	NCBI gi nimber 7435005	Non-Toxin	−0.9	0.69	0.21	−0.17	3.57	689.8
93	DEKPADL	0.19	allergen	NCBI gi nimber 1480457	Non-Toxin	−0.89	0.61	0.71	−0.35	4.03	786.92
94	VPAF	0.77	allergen	UniProtKB accession number O96522	Non-Toxin	−0.88	0.57	0	0.33	5.88	432.56
95	VPEDSQEECAIT	0.14	allergen	UniProtKB accession number P02636	Non-Toxin	−0.88	0.62	0.42	−0.19	3.51	1320.55
96	DVSI	0.07	allergen	UniProtKB accession number Q94507	Non-Toxin	−0.88	0.67	0	0.07	3.8	432.52
97	VNPH	0.20	allergen	NCBI gi nimber 41017429	Non-Toxin	−0.87	0.45	0.36	−0.14	7.1	465.56
98	GPVV	0.21	allergen	NCBI gi nimber 1311511	Non-Toxin	−0.86	0.61	0	0.29	5.88	370.5
99	DPDDF	0.81	allergen	NCBI gi nimber 110346534	Non-Toxin	−0.85	0.67	0	−0.32	3.43	607.62
100	SEEEDNEEEAEV	0.13	allergen	UniProtKB accession number P08334	Non-Toxin	−0.85	0.67	0.74	−0.43	3.32	1408.46
101	APNI	0.38	allergen	NCBI gi nimber 1174276	Non-Toxin	−0.84	0.58	0	0.07	5.88	413.52
102	QTL	0.35	allergen	UniProtKB accession number Q15517	Non-Toxin	−0.83	0.58	0.42	−0.11	5.88	360.45
103	PDL	0.41	allergen	UniProtKB accession number P09945	Non-Toxin	−0.82	0.55	0	−0.09	3.8	343.41
104	PTY	0.27	allergen	NCBI gi nimber 50199132	Non-Toxin	−0.82	0.53	0	−0.08	5.88	379.44
105	KPV	0.14	allergen	NCBI gi nimber 112559	Non-Toxin	−0.82	0.58	1.22	−0.21	9.11	342.47
106	TKP	0.13	allergen	UniProtKB accession number P86712	Non-Toxin	−0.82	0.52	1.22	−0.45	9.11	344.44
107	DKPVSP	0.19	allergen	UniProtKB accession number P10388	Non-Toxin	−0.81	0.57	0.61	−0.28	6.19	641.79
108	VNV	0.03	allergen	NCBI gi nimber 84698	Non-Toxin	−0.81	0.72	0	0.15	5.88	330.42
109	EVK	0.02	allergen	UniProtKB accession number Q1M2N1	Non-Toxin	−0.81	0.69	1.65	−0.39	6.35	374.47
110	GF	0.99	allergen	UniProtKB accession number P10385	Non-Toxin	−0.8	0.69	0	0.39	5.88	222.26
111	PSL	0.94	allergen	NCBI gi nimber 33323477	Non-Toxin	−0.8	0.47	0	0.07	5.88	315.4
112	IM	0.70	allergen	NCBI gi nimber 256636	Non-Toxin	−0.8	0.74	0	0.49	5.88	262.39
113	IG	0.50	allergen	NCBI gi nimber 208605348	Non-Toxin	−0.8	0.69	0	0.45	5.88	188.25
114	IPA	0.43	allergen	NCBI gi nimber 23616947	Non-Toxin	−0.8	0.53	0	0.3	5.88	299.4
115	AR	0.39	allergen	UniProtKB accession number P82901	Non-Toxin	−0.8	0.6	1.23	−0.76	10.11	245.29
116	GD	0.39	allergen	UniProtKB accession number P28508	Non-Toxin	−0.8	0.72	0	−0.28	3.8	190.17
117	PATIVPIDEESR	0.22	allergen	NCBI gi nimber 4097481	Non-Toxin	−0.8	0.6	0.42	−0.17	4.14	1326.63
118	IH	0.21	allergen	NCBI gi nimber 729979	Non-Toxin	−0.8	0.35	0.72	0.16	7.1	268.34
119	AA	0.19	allergen	UniProtKB accession number P86755	Non-Toxin	−0.8	0.52	0	0.25	5.88	160.18
120	NK	0.16	allergen	NCBI gi nimber 218059728	Non-Toxin	−0.8	0.72	1.83	−0.87	9.11	260.31
121	DH	0.15	allergen	UniProtKB accession number P20347	Non-Toxin	−0.8	0.38	0.72	−0.56	5.09	270.26
122	GEH	0.13	allergen	NCBI gi nimber 102834	Non-Toxin	−0.8	0.45	0.91	−0.29	5.25	341.36
123	ID	0.13	allergen	UniProtKB accession number Q94507	Non-Toxin	−0.8	0.73	0	0.01	3.8	246.28
124	GE	0.11	allergen	UniProtKB accession number P28508	Non-Toxin	−0.8	0.68	0.64	−0.23	4	204.2
125	SL	0.11	allergen	NCBI gi nimber 267048	Non-Toxin	−0.8	0.53	0	0.14	5.88	218.27
126	IK	0.10	allergen	NCBI gi nimber 157829757	Non-Toxin	−0.8	0.69	1.83	−0.19	9.11	259.37
127	TD	0.09	allergen	NCBI gi nimber 50199132	Non-Toxin	−0.8	0.65	0	−0.45	3.8	234.22
128	PD	0.09	allergen	NCBI gi nimber 4138171	Non-Toxin	−0.8	0.56	0	−0.4	3.8	230.23
129	TIDDGI	0.09	allergen	UniProtKB accession number P26213	Non-Toxin	−0.8	0.69	0	0	3.57	632.75
130	VH	0.07	allergen	UniProtKB accession number P16347	Non-Toxin	−0.8	0.35	0.72	0.07	7.1	254.31
131	NV	0.06	allergen	NCBI gi nimber 14423664	Non-Toxin	−0.8	0.73	0	−0.05	5.88	231.27
132	DK	0.06	allergen	UniProtKB accession number P00712	Non-Toxin	−0.8	0.72	1.83	−0.91	6.19	261.29
133	ED	0.03	allergen	UniProtKB accession number Q39858	Non-Toxin	−0.8	0.72	0.64	−0.67	3.67	262.23
134	VT	0.03	allergen	UniProtKB accession number Q17282	Non-Toxin	−0.8	0.61	0	0.18	5.88	218.27
135	VE	0.02	allergen	NCBI gi nimber 76097511	Non-Toxin	−0.8	0.69	0.64	−0.04	4	246.28
136	IDM	0.48	allergen	UniProtKB accession number Q94507	Non-Toxin	−0.79	0.75	0	0.09	3.8	377.49
137	GK	0.30	allergen	UniProtKB accession number P09944	Non-Toxin	−0.79	0.68	1.83	−0.47	9.11	203.26
138	DIPK	0.28	allergen	NCBI gi nimber 208605348	Non-Toxin	−0.79	0.62	0.92	−0.29	6.19	471.6
139	DATNVGDEGG	0.10	allergen	NCBI gi nimber 160347120	Non-Toxin	−0.79	0.68	0.13	−0.16	3.5	934.02
140	NVM	0.13	allergen	UniProtKB accession number P00777	Non-Toxin	−0.78	0.75	0	0.05	5.88	362.48
141	CQK	0.28	allergen	NCBI gi nimber 3318885	Non-Toxin	−0.77	0.66	1.64	−0.58	8.57	377.49
142	QSSASSPSPSK	0.27	allergen	NCBI gi nimber 886967	Non-Toxin	−0.76	0.53	0.45	−0.29	9.11	1062.23
143	DGK	0.21	allergen	UniProtKB accession number O60023	Non-Toxin	−0.76	0.71	1.22	−0.55	6.19	318.36
144	DGP	0.66	allergen	NCBI gi nimber 122064581	Non-Toxin	−0.74	0.6	0	−0.21	3.8	287.3
145	GPM	0.96	allergen	UniProtKB accession number P00974	Non-Toxin	−0.71	0.61	0	0.12	5.88	303.41
146	NGTI	0.15	allergen	NCBI gi nimber 18568322	Non-Toxin	−0.71	0.67	0	0.02	5.88	403.49
147	NGDK	0.94	allergen	UniProtKB accession number Q2YFF0	Non-Toxin	−0.69	0.72	0.92	−0.57	6.19	432.48
148	DQIDDEIK	0.13	allergen	NCBI gi nimber 47117350	Non-Toxin	−0.61	0.71	0.77	−0.39	3.84	975.13
149	DKPGPPAA	0.50	allergen	NCBI gi nimber 27806257	Non-Toxin	−0.55	0.53	0.46	−0.17	6.19	751.93
150	GVDNPGHP	0.42	allergen	NCBI gi nimber 285005081	Non-Toxin	−0.53	0.54	0.18	−0.13	5.09	791.93
151	ATGNPNPDIV	0.23	allergen	NCBI gi nimber 7489357	Non-Toxin	−0.42	0.61	0	−0.06	3.8	997.21
152	SGGTTMY	0.05	allergen	NCBI gi nimber 56417504	Non-Toxin	−0.38	0.63	0	0	5.88	715.87
153	PPGKPGPPGPPGPPGIQGIH	0.38	allergen	NCBI gi nimber 1168171	Non-Toxin	−0.09	0.5	0.32	−0.02	9.11	1854.45
154	IPGPPTGPIK	0.72	non-allergen	UniProtKB accession number Q9S8M0	Toxin	0.07	0.54	0.37	0.02	9.11	976.33

## 2 Experimental design, materials, and methods

### 2.1 Preparation and separation of chicken peptides

Peptides were produced and separated according to our previous study with slight modification [1]. In brief, chicken breast was digested in tris-HCl buffer (50 mM, pH 8.0, 1:5 m/v) by Alcalase 2.4L (0.5% w/w, protein basis) for 8 h. The hydrolysates were then centrifuged (10000 rpm, 20 min, 4 °C), and deproteinized (adding 3 volumes of ethanol, stored at 4 °C, 20h before centrifugation). After centrifugation at 10000 rpm at 4 °C for 20 min, the ethanol was removed in a rotatory evaporator, and the peptides were lyophilized and re-dissolved in distilled water for Sephadex G25 separation. The peptides were eluted using 0.01 N HCl at 4 °C with a flow rate of 5 mL/20 min. Fractions were collected, lyophilized and re-dissolved in distilled water for analysis and separation.

The most active fraction obtained from G25 fractionation was further isolated using HPLC with a Symmetry C18 column. The mobile phases consisted of solvent A: 0.1% v/v trifluoroacetic acetic acid (TFA) and solvent B: 0.085% v/v TFA in acetonitrile (ACN). Peptides were diluted using the following gradient: 0% B from 0 to 2 min, linearly increasing to 30% B at 50 min, 60% B at 60 min and 100% B at 65 min under a flow rate of 1 mL/min. Fractions were collected, lyophilized, and re-dissolved for analysis and identification.

### 2.2 Identification of peptide sequences

Peptides were identified using nano-LC tandem nanoelectrospray ionization source-quadrupole-time-of-flight (nanoESI-Q-ToF) MS/MS (AB Sciex Instruments, MA, USA). The sample was concentrated using Zip-Tip C18 (Millipore Corporation, Bedford, MA) before loaded onto an Eksigen trap column (3 μm C18-CL, 350 μm × 0.5 mm). Peptides were eluted using an analytical column (3 μm C18-CL, 75 μm × 123 mm; Nikkyo Technos Co, Ltd. Japan) under a flow rate of 0.3 μL/min at 30 °C. The mobile phases consisted of solvent A: 0.1% v/v formic acid (FA) in water and solvent B: 1% FA in acetonitrile (ACN). Peptides were eluted linearly from 5% to 35% solvent B over the first 20 min, and then from 35% to 65% solvent B for 10 min.

The flow from the LC was ionized applying 2.8 kV. The Q-ToF was operated in positive polarity and information-dependent acquisition mode. MS1 scan was acquired from 350 to 1250 *m*/*z* for 250 ms, while MS2 scan was required from 100 to 1500 *m*/*z* for 50 ms on 50 of the most intense ions charging from 1 to 5. Up to 25 ions were selected for fragmentation after each survey scan. Dynamic exclusion was set to 15 s.

The database searching of peptides was performed using the Mascot Distiller v2.4.2.0 software (Matrix Science, Inc., Boston, MA; http://www.matrixscience.com), and Mascot search engine with a significance threshold *p* < 0.05 using Chordata taxonomy, none enzyme digestion, and Uniprot database. The tolerance on the mass measurement was 0.3 Da for MS and 100 ppm for MS/MS.

### 2.3 *In silico* analysis of peptide bioactivity and applicability

*In silico* gastrointestinal digestion was assessed using the ExPASy PeptideCutter tool (http://web.expasy.org/peptide_cutter/). PeptideCutter predicts the potential cleavage sites by proteases in a given peptide sequence, according to the specific cleavage sites of proteases, and thus generate new peptides [2]. In present study, pepsin (pH 1.3 and pH > 2.0), trypsin, and chymotrypsin were chosen as digesting enzymes [3].

The potential bioactivity was predicted using the Peptide Ranker software (http://distilldeep.ucd.ie/PeptideRanker/) [4]. The prediction of peptide bioactivity was focused on particular amino acid residues as certain classes of bioactive peptides have specific structure features and amino acid sequences [5]. Peptides were scored from 0 to 1 and higher value means higher probability to be bioactive.

The potential peptide allergenicity was predicted using the AllerTOP v. 2.0 software (http://www.ddg-pharmfac.net/AllerTOP/index.html) [6]. Peptides were classified by k-nearest neighbor algorithm based on training set containing 2427 known allergens from different species and 2427 non-allergens.

Peptide toxicity and physicochemical properties (i.e., hydrophobicity, amphipathicity, steric hindrance, pI, and molecular weight) were studied using the ToxinPred software (http://crdd.osdd.net/raghava/toxinpred/) [7]. Peptide toxicity was predicted mainly according to the amino acid composition and position of peptides. The models were developed based on machine learning technique and quantitative matrix using more than 1805 toxic peptides.

## References

[1] Xiao C., Zhou F., Zhao M., Su G., Sun B. (2018). Chicken breast muscle hydrolysates ameliorate acute alcohol-induced liver injury in mice through alcohol dehydrogenase (adh) activation and oxidative stress reduction. Food Funct..

[2] Wilkins M.R., Gasteiger E., Bairoch A., Sanchez J.C., Hochstrasser D.F. (1999). Protein identification and analysis tools in the expasy server. Methods Mol. Biol..

[3] Garcia-Vaquero M., Mora L., Hayes M. (2019). In vitro and in silico approaches to generating and identifying angiotensin-converting enzyme i inhibitory peptides from green macroalga ulva lactuca. Mar. Drugs.

[4] Tu M., Liu H., Cheng S., Mao F., Chen H., Fan F., Lu W., Du M. (2019). Identification and characterization of a novel casein anticoagulant peptide derived from in vivo digestion. Food Funct..

[5] Mooney C., Haslam N.J., Pollastri G., Shields D.C. (2012). Towards the improved discovery and design of functional peptides: common features of diverse classes permit generalized prediction of bioactivity. PloS One.

[6] Polikovsky M., Fernand F., Sack M., Frey W., Müller G., Golberg A. (2019). In silico food allergenic risk evaluation of proteins extracted from macroalgae ulva sp. with pulsed electric fields. Food Chem..

[7] Gupta S., Kapoor P., Chaudhary K., Gautam A., Kumar R., Open S.D.D.C., Gajendra P.S.R. (2013). In silico approach for predicting toxicity of peptides and proteins. PloS One.

